# Obstetricians/Gynecologists' Problems in Sickness Certification Consultations: Two Nationwide Surveys

**DOI:** 10.1155/2016/9421316

**Published:** 2016-11-17

**Authors:** Catharina Gustavsson, Elin Hinas, Therese Ljungquist, Kristina Alexanderson

**Affiliations:** Department of Clinical Neuroscience, Division of Insurance Medicine, Karolinska Institutet, 171 77 Stockholm, Sweden

## Abstract

*Objective.* To explore experiences by physicians working in obstetrics, gynecology, or maternal healthcare (O/Gs) of problems in sickness certification consultations and differences between two years.* Material and Methods.* Answers by O/Gs to two Swedish nationwide surveys, in 2008 (*n* = 1037) and 2012 (*n* = 992), were analyzed for frequencies and severity of problems and organizational support in sickness certification consultations.* Results.* One-third of O/Gs found sickness certifications problematic every week. The most frequent problem was patients requesting sick notes for reasons other than work incapacity due to disease/injury (2008: 21%; 2012: 16%). The most problematic were assessing work capacity (2008 and 2012: 52%) and having different opinion from that of the patient about need for sick leave (2008: 51%; 2012: 46%). In 2012, 27% used the national sickness certification guidelines weekly, compared to 9% in 2008. A larger proportion in 2012 than 2008 reported that the guidelines facilitated contacts with patients and different stakeholders.* Conclusions.* Although O/Gs perceived sickness certification as problematic, there was less perceived severity of problems in 2012 compared to 2008, possibly because interventions regarding sickness certification have been introduced in Sweden recent years. Still, more organizational support, for example, time and supervision, are needed to enhance O/Gs' sickness certification practices.

## 1. Introduction

Sickness certification consultations constitute a complex work task for physicians in most western countries [1, 2]. In Sweden, after seven days of self-certification, sickness absentees need a medical certificate issued by a physician when claiming sick-pay from employer or sickness benefits from the Social Insurance Agency (SIA). These certificates are the bases for the decision from the SIA on whether the patient fulfils the criteria for sickness benefits or not [3]. The physicians' competence in insurance medicine and how physicians handle sickness certifications consultations can have considerable impact on sick-leave levels [1]. Despite the great impact for patients and society, the knowledge on physicians' sickness certification practice is limited [1, 4]. Studies have mainly targeted general practitioners [5, 6]. Few studies comprise physicians in other clinical settings [4, 7]. In two large nationwide surveys of physicians' sickness certifications, in 2008 [7] and 2012 [8], we collected data from physicians in most clinical settings.

In a previous study [9], answers from physicians working in obstetrics, gynecology, or maternal healthcare (here called O/Gs) in two Swedish counties in 2004 and 2008 were explored. We found that the O/Gs often perceived sickness certifications as problematic. However, that study covered only O/Gs in two counties, that is, only about one-fifth, of all Swedish O/Gs. In the present study, we could include all O/Gs working in Sweden, likewise exploring changes over time, since data from 2012 was available and could be compared to 2008.

After 2008, several interventions have been undertaken in Sweden, to facilitate physicians' sickness certification practice: foremost educational efforts, that is, courses in insurance medicine and accessibility to expertise in insurance medicine, but also promoting support at an organizational level through, for example, introducing workplace policies at clinical levels for this [10]. Few studies have evaluated the effects of such interventions, and to the best of our knowledge, no study has specifically targeted O/Gs. One study showed that physicians having a well-established workplace policy regarding sickness certification matters found assessing long-term prognosis of work capacity less problematic [11]. In a Norwegian intervention, general practitioners who had participated in training regarding sickness certification reported improved knowledge of and self-efficacy for carrying out the functional assessments related to the sickness certificate [12]. Also, in 2007, the Swedish Board of Health and Welfare for the first time introduced national general and diagnoses-specific sickness certification guidelines [13]. The purpose was to provide a facilitating framework and ensure safe and equal sickness certification of high quality for the patient.

For O/Gs, there are specific aspects to consider in sickness certifications tasks, first; nearly all their patients are women, and women have higher sick-leave rates, they often have a work situation which differs from that of men [14, 15], and sickness certification of pregnant women involves some special concerns [16]. In Sweden, sickness absence among pregnant women is common, despite the fact that the social insurance system comprises the possibility of attaining pregnancy or parental benefits during the last trimester [16–19]. Sickness certification consultations might propose delicate handling by the treating physicians, as to determine if the pregnancy impairs the patient's function to the extent that work capacity is also impaired in relation to the demands of the patient's work [20, 21]. To the best of our knowledge, there is only one study on how O/Gs experience sickness certification of pregnant women [22], showing several types of problematic situations. Thus, more knowledge is warranted on this issue, as bases for interventions to facilitate O/Gs' sickness certification practices and ensure optimal sickness certification for their patients.

The objective of this study was to explore experiences by physicians working in obstetrics, gynecology, or maternal healthcare (O/Gs) of problems in sickness certification consultations regarding (i) frequencies and perceived severity of problems related to such tasks; (ii) use of sickness certification guidelines and other means to ensure high quality in sickness certifications; (iii) the need to acquire more competence regarding sickness certification issues; and (iv) differences in responses between two nationwide surveys in 2008 and 2012.

## 2. Methods

Data were obtained from two cross-sectional questionnaire studies of sickness certification practice, mail administered at two occasions; in October 2008 to all physicians living and working in Sweden (*n* = 36 898) [7] and in October 2012 to all physicians in clinical settings where sickness certification occurred and aged <68 years (*n* = 31 959). The study populations were identified using a register of all physicians in Sweden (Cegedim Sweden AB) including information about age, sex, and type of board-certified specialist qualification from the National Board of Health and Welfare. These studies were approved by the Regional Ethical Review Board of Stockholm, Sweden (Reg. numbers 2008/795-31, 2012/689-31/5). The response rates were 61% in 2008 and 58% in 2012.

In this study, responses from physicians <68 years old and mainly working in obstetrics, gynecology, or maternal healthcare were included. In 2008, 1037 physicians and, in 2012, 992 physicians met these criteria (Table 1).

The 2008 questionnaire included 163 questions concerning various aspects of the physician's sickness certification consultations, based on previous studies [7, 23]. The 2012 questionnaire had the same number of questions but some had been removed and others were added; beyond this, the wording of a few questions was elaborated to be less ambiguous. In this study, only questions included in both surveys were examined. Answers to the following questions were analyzed.

*Frequency of sickness certification consultations* was measured by the question “How often in your daily clinical work do you have consultations including consideration of sickness certification?” with response options “more than 20 times a week,” “6–20 times a week,” “1–5 times a week,” “a few times a month,” “a few times a year,” and “never or almost never.” In analyses, the response options “more than 20 times a week” and “6–20 times a week” were merged to “at least 6 times per week” and “a few times a month” and “a few times a year” were merged to “a few times per month or year.” In order to focus on O/Gs with frequent experiences of such consultations, those responding with “a few times a month,” “a few times a year,” or “never or almost never” were excluded in the further analysis.

*Frequency of problematic situations in sickness certification consultations* was measured by the overall question “How often in your clinical work do you…” followed by descriptions of different situations, with response options: “more than 10 times a week,” “6–10 times a week,” “1–5 times a week,” “a few times a month,” “a few times a year,” and “never or almost never.” In the analyses, the response options “more than 10 times a week,” “6–10 times a week,” and “1–5 times a week” were merged to “at least once a week.” The response options “a few times a month” and “a few times a year” were merged to “a few times per month or year.”

*Severity of problems regarding sickness certification consultations* was measured by the overall question “How problematic do you generally find it to…” followed by descriptions of eighteen situations, with response options “very,” “fairly,” “somewhat,” and “not at all.” Three of the situations also had the additional response option “not applicable.”

*Lack of time when handling sickness certification tasks* was measured by three questions “When handling sickness certification tasks, how often do you experience lack of time… (i) with your patient? (ii) to manage patient-related aspects? (iii) for further education, supervision or reflection?”, with response options: “every day,” “about once a week,” “about once a month,” “a few times a year,” and “never or almost never.”

*Use and usefulness of the national sickness certification guidelines* were measured by the questions “How often in your clinical work do you apply the national sickness certification guidelines?” (response options: “at least once a week,” “a few times per month or year,” and “never or almost never”) and “Do the new national sickness certification guidelines provided by the National Board of Health and Welfare facilitate your contacts with… (i) your patient? (ii) healthcare staff? (iii) patients' workplace/the Employment Office? (these two questions were merged in the analysis because they were two separate questions in 2012 but were combined to one question in 2008) (iv) Social Insurance Agency?” (response options “yes,” “no”).

*Value of means/resources to ensure high quality in sickness certification cases* was measured by the overall question “How do you value the following options with regard to ensure high quality of your handling of sickness certification cases?”, followed by descriptions of fourteen means/resources, with response options “very beneficial,” “moderately beneficial,” and “not beneficial.”

*Need to acquire more competence regarding issues related to sickness certification* was measured by the overall question “To what extent do you need to further develop your competence regarding the following…” followed by descriptions of seventeen situations, with response options “to a large extent,” “to a fairly large extent,” “to a small extent,” and “not at all.” In the analysis, the response options “to a large extent” and “to a fairly large extent” were merged to “to a large/fairly large extent.”

Data was analyzed with descriptive statistics. Only the O/Gs who reported that they had sickness certification consultations at least once a week were included in the further analyses of experiences in regard to sickness certification issues. The Mann–Whitney *U* test was applied to evaluate differences between years. A *p* value ≤ 0.05 was accepted as statistically significant. Analyses were conducted using the statistical software SPSS 20.0.

## 3. Results

A majority of the responding physicians working in gynecology, obstetrics, or maternal healthcare (O/Gs) were women, 45–67 years old, and board-certified specialists in gynecology/obstetrics (Table 1). Corresponding information regarding O/Gs who did not respond to the surveys was not available. However, information about nonresponders was available for all board-certified O/Gs, regardless of which type of clinic they worked in. Response rate to the surveys among board-certified O/Gs was somewhat higher than for all physicians: in 2008 their response rate was 66.9% and in 2012 63.6%. For 2012, information on age and gender for board-certified O/Gs was available: among nonresponders, the proportions of women and older physicians were somewhat lower than those among responding O/Gs.

The further analyses include only O/Gs that reported having sickness certification consultations at least once a week: that is, 739 (71.3%) in 2008 and 662 (66.8%) in 2012.

Frequencies of problematic situations in sickness certification consultations perceived by those O/Gs are presented in Table 2. In both years, nearly one-third experienced such consultations as problematic at least once a week. The most frequently occurring problematic situation was to “…encounter a patient who wants to be on sick leave for some reason other than work incapacity due to disease or injury,” reported occurring weekly by 21.1% in 2008 and by 16.0% in 2012. Almost all O/Gs (90.7% in 2008 and 92.5% in 2012) at least a few times per year or month said no to a patient who wanted a sickness certificate; this was the only frequency that was higher in 2012 than in 2008. All other listed problematic situations were reported by a larger proportion of O/Gs in 2008 compared to in 2012. For instance, 78.1% in 2008 and 72.8% in 2012 reported having conflicts with patients about sickness certification at least a few times per year or month (*p* = 0.009).

The perceived severity of different problems in sickness certification is presented in Table 3. Both years, about half of the O/Gs found it very or fairly problematic (51.8% in 2004 and 51.6% in 2008) to “assess the degree to which the reduced functional capacity limits a patient's work capacity” and to “handle situations in which you and a patient have different opinions about the need for sick leave” (2008: 51% and 2012: 46%). Other situations perceived as very or fairly problematic by a large proportion of the O/Gs were to “decide whether to certify prolongation of a sick-leave period initially certified by another physician,” “assess the optimum duration and degree of sickness absence,” and “assess whether a patient's functional capacity is reduced.” In 2012, a smaller proportion reported these situations as very or fairly problematic, as compared to in 2008.

When handling sickness certification tasks, a majority experienced lack of time at least once a week in relation to time with the patient (in 2008 56.8% and in 2012 60.1%), time to manage patient-related aspects (in 2008 56.1% and in 2012 63.1%), and time for further education, supervision, or reflection (in 2008 52.3% and in 2012 52.0%).

**Figure 1:**
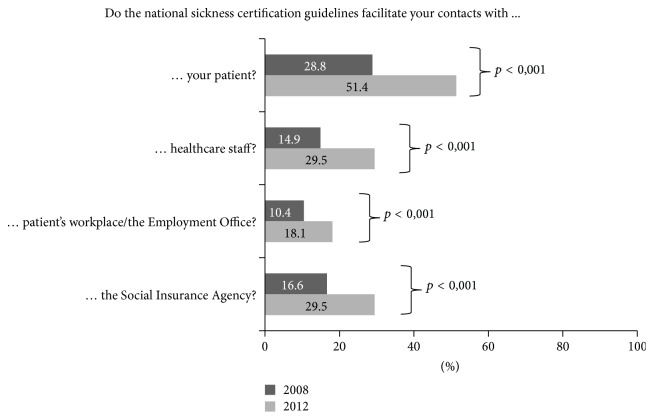
Proportions (%) of O/Gs reporting that the national sickness certification guidelines facilitated their contacts with the patient or different stakeholders in sickness certification consultations, in 2008 (*n* = 739) and in 2012 (*n* = 662), respectively, and *p* values for significance level of differences between 2008 and 2012.

In 2012, 26.7% of the O/Gs reported that they used the national sickness certification guidelines every week, as compared to 8.7% in 2008. In 2012, 26.4% responded that they never used the guidelines, as opposed to 52.5% in 2008. In 2012, half of the O/Gs (51.4%) responded that the national sickness certification guidelines facilitated their contacts with patients, as opposed to less than a third (28.8%) in 2008 (Figure 1).

**Figure 2:**
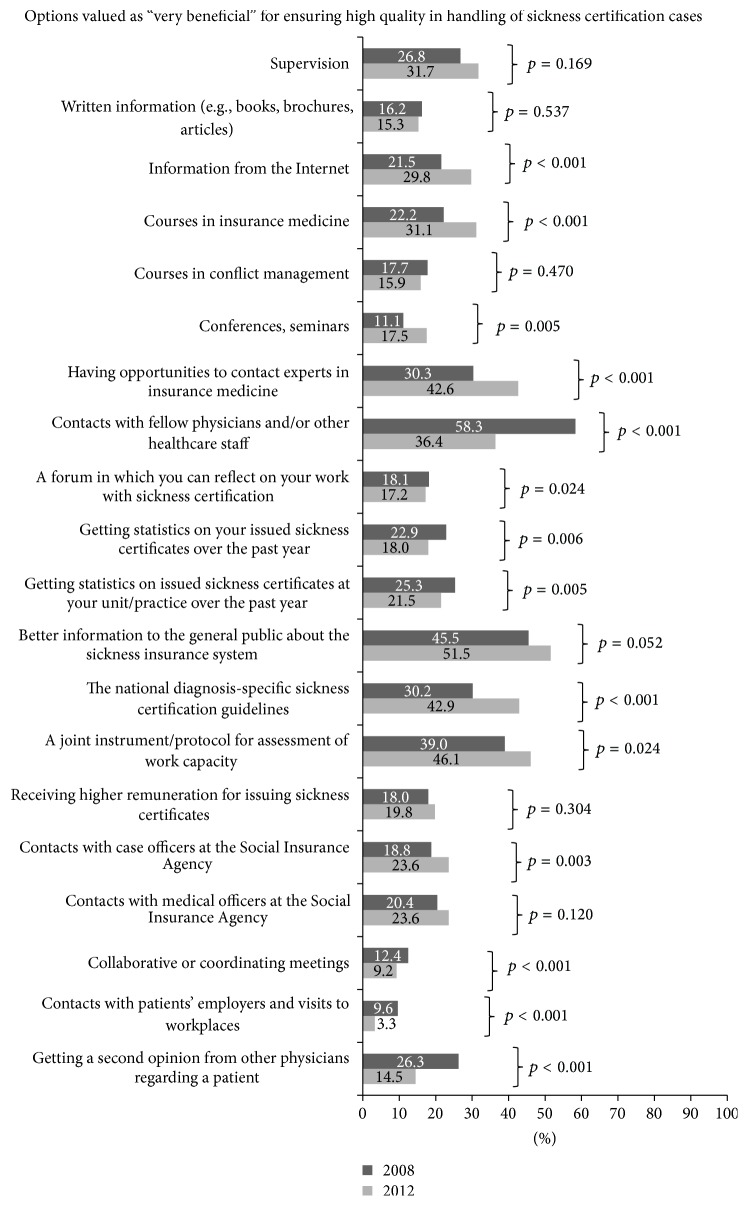
Proportions (%) of O/Gs reporting different options to be very beneficial in order to ensure high quality in handling sickness certification cases, in 2008 (*n* = 739) and 2012 (*n* = 662), respectively, and *p* values for differences between 2008 and 2012.

Responses regarding finding listed options to be “very beneficial” for ensuring high quality in sickness certification are shown in Figure 2. For many of the options, there were differences between the years. In 2008, the options valued as “very beneficial” by the largest proportion of O/Gs were “contact with fellow physicians and/or other health care staff” (58.3%) and “better information to the general public about the sickness insurance system” (45.5%). The latter option was valued as “very beneficial” by the largest proportion of O/Gs (51.5%) in 2012, followed by the option “a joint instrument/protocol for assessment of work capacity” (46.1%). The option that was valued as “very beneficial” by the smallest proportion in both 2008 (9.6%) and 2012 (3.3%) was “contacts with patients” employers and visits to workplaces.

**Figure 3:**
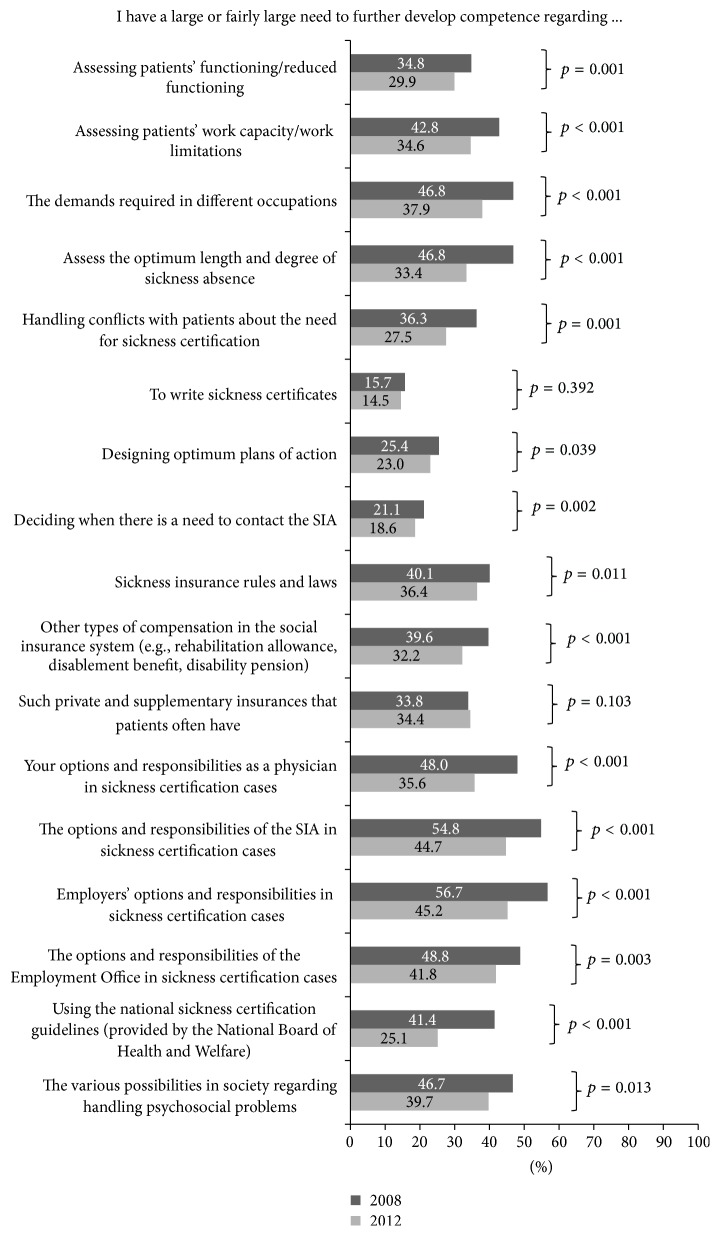
Proportions (%) of O/Gs reporting large or fairly large need to further develop their competence regarding different aspects regarding sickness certification issues, in 2008 (*n* = 739) and 2012 (*n* = 662), respectively, and *p* values for differences between 2008 and 2012.

A significantly smaller proportion of O/Gs in 2012 compared to in 2008 responded that they had a large/fairly large need for more competence in sickness certification issues concerning most of the listed options (Figure 3). In 2012, the response options that the largest proportion reported a large/fairly large need to further develop their competence were the employers' (45.2%), Social Insurance Agency's (44.7%), and Employment Office's (41.8%) options and responsibilities in sickness certification cases.

## 4. Discussion

In this first nationwide study of the physicians working in obstetrics, gynecology, or maternal healthcare regarding their experiences of sickness certification tasks, we found that a vast majority of the responding O/Gs had sickness certification consultations every week and that almost one-third experienced sickness certification as problematic at least once a week. The most frequent problematic situation was to handle patients requesting sickness certificates for some other reason than work incapacity due to disease or injury. However, most types of problems were less frequent in 2012, indicating that fewer O/Gs perceived problems in 2012 compared to 2008. It could be hypothesized that the interventions undertaken in between the two surveys to facilitate sickness certification practice [10] have contributed to more competence in insurance medicine among O/Gs. Still, more organizational support and administrative resources, for example, time, training, and supervision, are needed to further enhance O/Gs sickness certification practice.

Regarding severity of problems, large proportions of O/Gs found several aspects of accessing work capacity as problematic and many wanted an instrument for such assessments. The same tendency was found in a previous survey that explored O/Gs in two Swedish counties in 2004 and 2008 [9] and in a smaller survey by Larsson et al. [22]. Specifically, half of O/Gs found it problematic to assess the degree to which the reduced functional capacity limits a patient's work capacity. Also, almost half of the O/Gs stated that an instrument/protocol for assessment of work capacity would be very beneficial to ensure high quality in sickness certification cases. A higher proportion responded this in 2012 than in 2008. Our results are in line with studies of physicians from other specialties, showing that assessing the patient's work capacity is problematic in sickness certifications [21, 24]. This is an important area to highlight in further interventions aiming at improving competence and provide organizational support.

It is notable that contacts with patients' employers and workplaces were valued as beneficial by the smallest proportion in both surveys. It has been argued that contacts with employers and workplaces could highlight and bring forward measures to facilitate for employees to remain at work and avoid long-term sick leave [25]. However, few physicians have trainings on how to handle such contacts and little time to take part in them. In Sweden, it is currently discussed how to promote such contacts and to what extent other healthcare professionals can handle some of them. For instance, the so-called sick-leave coordinators are introduced in clinical settings to promote such contacts [26]. More research is needed on effects of that.

A majority experienced lack of time at least once a week regarding sickness certification tasks. These figures remained fairly stable between the two years. However, in 2012, a larger proportion of O/Gs experienced lack of time every week to manage patient-related aspects, such as documentation and contacts with other stakeholders, compared to 2008. This is a perturbing development that needs to be addressed at organizational levels in healthcare [27].

In 2012, one-fourth of O/Gs reported using the national sickness certification guidelines weekly, as compared to only 8.7% in 2008. In comparison, a study of general practitioners showed that, already in 2008, 18% used the guidelines weekly [13]. In 2012, half of the O/Gs responded that the sickness certification guidelines facilitated their contacts with patients, as opposed to less than a third in 2008. Moreover, a larger proportion in 2012 than 2008 reported that the guidelines facilitated contacts with healthcare staff, SIA staff, and the patient's workplace. It is not surprising that these guidelines had not gained common ground in 2008, since they were introduced in the late 2007, and thus had only been in use for a year. New guidelines usually take longer time to become implemented in healthcare [28]. It is an important finding that they were in use in clinical practice in 2012 and perceived as useful.

In between these surveys, several educational interventions were initiated (e.g., courses and accessibility to expertise in insurance medicine) [10, 27]. Only a few studies have evaluated the effects of such interventions and have suggested that well-established workplace policies and education improve physicians' competence and self-efficacy for sickness certification tasks in Sweden, as well as in other countries [11, 12]. Although a somewhat smaller proportion of O/Gs in 2012 than 2008 responded that they needed to acquire more competence in issues related to sickness certifications, the results indicate that additional interventions are needed. In 2012, almost half of the O/Gs reported a need to develop their competence in insurance medicine, for example, regarding the employers', the SIA's, and Employment Office's responsibilities in sickness certification cases. In Sweden, it has also been underscored that these issues need to be addressed at healthcare management levels, by, for example, supportive leadership, workplace policies, and supervision [27, 29].

Strengths of this study are the fact that all O/Gs working in Sweden were included, the high response rates, and the fact that data from surveys in two different years was included. To our knowledge, this is the first study concerning O/Gs' sickness certification practice based on such large study groups. Further, the comprehensive questionnaires gave the possibility to study aspects at detailed levels. A limitation is that background information on nonresponders was not available specifically for* all* physicians working in gynecology, obstetrics, or maternal healthcare (O/Gs), only for board-certified O/Gs. Hence, bias to nonresponders cannot be ruled out. A possible limitation is that comparison between the years might be affected by differences between the two study groups. Apart from gender (i.e., a larger proportion of women in 2012 than 2008), there were no significant differences between the two study groups.

## 5. Conclusion

Our findings show that O/Gs frequently found sickness certification tasks problematic, although they perceived a somewhat lower severity of problems in 2012 compared to 2008. This change might be related to interventions undertaken in Sweden in between the two surveys, such as guidelines and training possibilities to enhance insurance medicine competence and practice development. The most frequently occurring problematic situation for O/Gs was handling patients requesting sickness certificates for other reasons than work incapacity due to disease or injury. The O/Gs found it especially problematic to assess the patient's work capacity and expressed the need of instruments for such assessments. Thus, more scientific knowledge on sickness absence and sickness certification in these patient groups is warranted. Also, more organizational support and administrative resources for O/Gs are needed: especially more time, not only for patient contacts but also for training, supervision, and contacts with other stakeholders, and to keep updated on guidelines. Further, O/Gs also need to have access to dialogue with special competences, that is, expertise in insurance medicine matters. Such resources and competence are needed to enhance O/Gs' sickness certification practices.

## Figures and Tables

**Table 1:** Characteristics of the O/Gs participating in the surveys 2008 and 2012, respectively, and frequency of sickness certification consultations by sex, age group, and type of specialist certification.

			Frequency of sickness certification consultations				
		Participants	≥6 times per week	1–5 times per week	A few times per month or year	Never or almost never	Missing information
		*n*	*n* (%)	*n* (%)	*n* (%)	*n* (%)	*n* (%)

	2008	1037	284 (27.4)	455 (43.9)	175 (16.9)	117 (11.3)	6 (0.6)
	2012	992	186 (18.8)	476 (48.0)	214 (21.6)	114 (11.5)	2 (0.2)

		m/md/min-max					

	Age 2008	48.9/50/25–67					
	Age 2012	48.8/49/27–67					

		*n* (%)					

2008	Women	693 (66.8)	203 (29.3)	314 (45.3)	98 (14.1)	75 (10.8)	3 (0.4)
	Men	344 (33.2)	81 (23.5)	141 (41.0)	77 (22.4)	42 (12.2)	3 (0.9)
2012	Women	700 (70.6)	145 (20.7)	356 (50.9)	132 (18.9)	66 (9.4)	1 (0.1)
	Men	292 (29.4)	41 (14.0)	120 (41.1)	82 (28.1)	48 (16.4)	1 (0.3)

2008	25–44 ys	389 (37.5)	127 (32.6)	222 (57.1)	34 (8.7)	6 (1.5)	0 (0.0)
	45–67 ys	648 (62.5)	157 (24.2)	233 (36.0)	141 (21.8)	111 (17.1)	6 (0.9)
2012	27–44 ys	392 (39.5)	87 (22.2)	237 (60.5)	65 (16.6)	3 (0.8)	0 (0.0)
	45–67 ys	600 (60.5)	99 (16.5)	239 (39.8)	149 (24.8)	111 (18.5)	2 (0.3)

	Specialist^a^	800 (77.1)	210 (26.2)	324 (40.5)	146 (18.2)	115 (14.4)	5 (0.6)
2008	Specialist other^b^	10 (1.0)	2 (20.0)	4 (40.0)	1 (10.0)	2 (20.0)	1 (10.0)
	Nonspecialist	227 (21.9)	72 (31.7)	127 (55.9)	28 (12.3)	0 (0.0)	0 (0.0)
	Specialist^a^	766 (77.2)	145 (18.9)	338 (44.1)	172 (22.5)	109 (14.2)	2 (0.3)
2012	Specialist other^b^	10 (1.0)	1 (10.0)	5 (50.0)	0 (0.0)	4 (40.0)	0 (0.0)
	Nonspecialist	216 (21.8)	40 (18.5)	133 (61.6)	42 (19.4)	1 (0.5)	0 (0.0)

^a^Physicians in study groups having board specialist certification in obstetrics and/or gynecology.

^b^Physicians in study groups having board specialist certification in specialties other than obstetrics and gynecology.

**Table 2:** Proportions (%) of O/Gs (having sickness certifications at least weekly) reporting frequencies of different situations in sickness certification consultations in 2008 (*n* = 739) and in 2012 (*n* = 662), respectively, and *p* values for differences between 2008 and 2012.

How often in your clinical work…	Year	At least once a week	A few times per month or year	Never or almost never	Missing information	Between-group comparison
		*n* (%)	*n* (%)	*n* (%)	*n* (%)	*p* value^a^
…do you find it problematic to handle sickness certifications?	2008	234 (31.7)	459 (62.1)	38 (5.1)	8 (1.1)	0.367
	2012	202 (30.5)	421 (63.6)	37 (5.6)	2 (0.3)	
…do you encounter a patient who wants to be on sick leave for some other reason than work incapacity due to disease or injury?	2008	156 (21.1)	487 (65.9)	82 (11.1)	14 (1.9)	0.001
	2012	106 (16.0)	450 (68.0)	101 (15.3)	5 (0.8)	
…do patients say no, partly or completely, to a sick leave you suggest?	2008	51 (6.9)	457 (61.8)	221 (29.9)	10 (1.4)	0.001
	2012	37 (5.6)	371 (56.0)	251 (37.9)	3 (0.5)	
…do you say no to a patient who wants a sickness certificate?	2008	79 (10.7)	591 (80.0)	50 (6.8)	19 (2.6)	0.078
	2012	62 (9.4)	550 (83.1)	47 (7.1)	3 (0.5)	
…do you experience conflicts with patients about sickness certification?	2008	71 (9.6)	506 (68.5)	151 (20.4)	11 (1.5)	0.009
	2012	57 (8.6)	425 (64.2)	175 (26.4)	5 (0.8)	
…do you worry that a patient will report you to the medical disciplinary board regarding sickness certification?	2008	11 (1.5)	92 (12.4)	630 (85.3)	6 (0.8)	0.311
	2012	5 (0.8)	78 (11.8)	576 (87.0)	3 (0.5)	
…do you feel threatened by a patient in connection with sickness certification?	2008	7 (0.9)	108 (14.6)	615 (83.2)	9 (1.2)	0.041
	2012	2 (0.3)	73 (11.0)	577 (87.2)	10 (1.5)	
…do you issue sickness certificates to patients without seeing them (e.g., by telephone)?	2008	40 (5.4)	495 (67.0)	196 (26.5)	8 (1.1)	0.612
	2012	29 (4.4)	456 (68.9)	175 (26.4)	2 (0.3)	
…do you worry that patients will change physician if you don't issue a sickness certificate?	2008	8 (1.1)	61 (8.3)	665 (90.0)	5 (0.7)	0.630
	2012	7 (1.1)	48 (7.3)	601 (90.8)	6 (0.9)	
…do patients say that they will change physician if you don't issue a sickness certificate?	2008	3 (0.4)	118 (16.0)	610 (82.5)	8 (1.1)	0.005
	2012	2 (0.3)	74 (11.2)	582 (87.9)	4 (0.6)	

^a^Mann–Whitney *U *test.

**Table 3:** Number and proportions (%) of O/Gs reporting severity of problems, that is, how problematic they found specific situations in sickness certification consultations in 2008 (*n* = 739) and in 2012 (*n* = 662), respectively, and *p *values for differences between 2008 and 2012.

How problematic do you generally find it to…	Year	Very	Fairly	Somewhat	Not at all	Not applicable	Missing information	Between-group comparison
		*n* (%)	*n* (%)	*n* (%)	*n* (%)	*n* (%)	*n* (%)	*p* value^a^
…handle sickness certification of patients?	2008	36 (4.9)	198 (26.8)	369 (49.9)	121 (16.4)		15 (2.0)	0.648
	2012	30 (4.5)	182 (27.5)	342 (51.7)	97 (14.7)		11 (1.7)	
…assess whether a patient's functional capacity is reduced?	2008	70 (9.5)	242 (32.7)	303 (41.0)	101 (13.7)		23 (3.1)	**0.016**
	2012	50 (7.6)	187 (28.2)	309 (46.7)	101 (15.3)		15 (2.3)	
…assess whether the reduced functional capacity is due to disease/injury?	2008	47 (6.4)	189 (25.6)	344 (46.5)	140 (18.9)		19 (2.6)	**0.018**
	2012	42 (6.3)	140 (21.1)	304 (45.9)	159 (24.0)		17 (2.6)	
…assess the degree to which the reduced functional capacity limits a patient's work capacity?	2008	122 (16.5)	261 (35.3)	288 (39.0)	55 (7.4)		13 (1.8)	0.241
	2012	85 (12.8)	257 (38.8)	238 (36.0)	69 (10.4)		13 (2.0)	
…consider, together with the patient, the advantages and disadvantages of being on sick leave?	2008	38 (5.1)	159 (21.5)	337 (45.6)	189 (25.6)		16 (2.2)	0.137
	2012	23 (3.5)	143 (21.6)	290 (43.8)	194 (29.3)		12 (1.8)	
…make a plan of action regarding measures to be taken during the sick leave?	2008	47 (6.4)	145 (19.6)	276 (37.3)	252 (34.1)		19 (2.6)	0.520
	2012	46 (6.9)	121 (18.3)	232 (35.0)	239 (36.1)		24 (3.6)	
… provide a long-term prognosis about the future work capacity of patients on sick leave?	2008	80 (10.8)	132 (17.9)	266 (36.0)	240 (32.5)		21 (2.8)	0.848
	2012	70 (10.6)	130 (19.6)	209 (31.6)	227 (34.3)		26 (3.9)	
…manage the two roles as the patient's treating physician and as a medical expert for the SIA and other authorities?	2008	98 (13.3)	164 (22.2)	277 (37.5)	183 (24.8)		17 (2.3)	0.660
	2012	65 (9.8)	170 (25.7)	244 (36.9)	165 (24.9)		18 (2.7)	
… discuss possible lifestyle and life situation changes with a patient who is being issued a sickness certificate?	2008	49 (6.6)	191 (25.8)	295 (39.9)	184 (24.9)		20 (2.7)	**0.009**
	2012	37 (5.6)	143 (21.6)	254 (38.4)	201 (30.4)		27 (4.1)	
…discuss and know how to deal with other psychosocial problems (e.g., economic difficulties, physical or substance abuse) when handling a patient on sick leave?	2004	85 (11.5)	204 (27.6)	271 (36.7)	158 (21.4)		21 (2.8)	0.080
	2008	65 (9.8)	170 (25.7)	234 (35.3)	166 (25.1)		27 (4.1)	
…know what aspects of the sickness certification process to document in the patient's medical file?	2008	28 (3.8)	99 (13.4)	296 (40.1)	299 (40.5)		17 (2.3)	0.441
	2012	25 (3.8)	108 (16.3)	248 (37.5)	262 (39.6)		19 (2.9)	
…decide whether to certify a prolongation of a sick-leave period initially certified by another physician?	2008	98 (13.3)	252 (34.1)	269 (36.4)	106 (14.3)		14 (1.9)	**0.014**
	2012	63 (9.5)	198 (29.9)	290 (43.8)	93 (14.0)		18 (2.7)	
…assess the optimum duration and degree of sickness absence?	2008	90 (12.2)	256 (34.6)	303 (41.0)	75 (10.1)		15 (2.0)	**<0.001**
	2012	53 (8.0)	200 (30.2)	302 (45.6)	289 (13.4)		18 (2.7)	
…handle situations in which you and a patient have different opinions about the need for sick leave?	2008	129 (17.5)	250 (33.8)	263 (35.6)	84 (11.4)		13 (1.8)	0.050
	2012	87 (13.1)	218 (32.9)	264 (39.9)	76 (11.5)		17 (2.6)	
…write sickness certificates for the SIA?	2008	49 (6.6)	155 (21.0)	309 (41.8)	205 (27.7)		21 (2.8)	0.376
	2012	44 (6.6)	128 (19.3)	257 (38.8)	197 (29.8)		36 (5.4)	
…write other certificates for the SIA? (e.g. for application concerning disability pension)	2008	82 (11.1)	229 (31.0)	237 (32.1)	151 (20.4)	—	40 (5.4)	—
	2012	29 (4.4)	47 (7.1)	62 (9.4)	37 (5.6)	463 (69.9)^b^	24 (3.6)	
…write sickness certificates in accordance with the national sickness certification guidelines?	2008	18 (2.4)	74 (10.0)	171 (23.1)	63 (8.5)	395 (53.5)^c^	18 (2.4)	—
	2012	46 (6.9)	148 (22.4)	250 (37.8)	152 (23.0)	—	66 (10.0)	
…handle situations in which you and other members of the healthcare team have different opinions about sickness certifying a patient?	2008	26 (3.5)	85 (11.5)	155 (21.0)	114 (15.4)	347 (47.0)^b^	12 (1.6)	**0.002**
	2012	16 (2.4)	43 (6.5)	124 (18.7)	109 (16.5)	346 (52.3)^b^	24 (3.6)	

^a^Mann–Whitney *U* test. ^b^An additional response option “not applicable” was used for three questions.

^c^The additional response option was phrased as “have not used them.”

## Abbreviations

O/Gs: Physicians working in obstetrics, gynecology, or maternal healthcare.
